# Understanding fire regimes: A biogeographical perspective

**DOI:** 10.4102/jamba.v16i1.1673

**Published:** 2024-07-26

**Authors:** Daniel S. Boshoff

**Affiliations:** 1 Disaster Risk Science, Unit for Environmental Science and Management, Faculty of Natural and Agricultural Science, North-West University, Vanderbijlpark, South Africa

**Keywords:** fire regimes, fire drivers, fire event, fire characteristics, fire risk management, fire ecology, wildfire studies

## Abstract

Fire regimes are often considered to be either driven by climate, fuel load or human activities. A significant proportion of fires across various ecosystems occur via large fire events. Recently, suggestions have been made that fires are becoming more severe and frequent as a consequence of current climate change. Although there are many factors influencing fire events, scientists have not found a suitable framework that can provide for understanding fires at the macroscale level. This review article proposes a new conceptual framework to better understand fire regimes. The proposed framework relies on a biogeographical perspective of fire regimes that include characteristics that have been underestimated in previous frameworks and to mitigate time as well as spatial scale issues at the macrolevel.

## Introduction

Naturally, wildfires have existed all over the Earth’s land surface before humans started influencing them. Fires are essential for clearing plant debris and enabling the regeneration of specific plant species (Fidelis 2020). Additionally, fires affect plants by promoting resprouting, germination and flowering after they occur (Midgley & Bond 2013). Fires impact ecosystems globally, regardless of their ignition source – natural or human induced. Moreover, fires pose significant threats to human lives, health and livelihoods (Edwards et al. 2020). For instance, one fire event in South Africa’s Garden Route region caused the deaths of seven people, resulted in approximately USD 105 million in property damages and burned down more than 5000 ha of forest (Kraaij et al. 2018; Forsyth et al. 2018; Frost et al. 2018). According to NASA (2023), the Northern Hemisphere experienced an increased frequency of fires and a prolonged fire season in 2023 with large fires occurring in Canada, Greece, Hawaii and the Canary Islands. It is argued that warmer temperatures from climate change are mostly responsible for the unusual fire season (NASA 2023). However, conditions favouring the spread of fires are dynamic and multifaceted. Grasping the behaviour, spread and occurrence of fire is a complex endeavour. In fact, it has been argued that ‘a case can be made for fire being, next to life processes, the most complex of phenomena to understand’ (Rein 2013:16). The occurrence of fires is dependent on flammable materials, sources of ignition and the climate (Holsinger, Parks & Miller 2016). Nevertheless, it is important to look past simplifications and generalisations of fire occurrences and to focus on the specific characteristics and drivers of fires to truly understand the patterns of these events over large temporal and spatial scales. This can be crucial to informing disaster risk planning and fire management plans, strategies and policies. Studying fires requires knowledge of fire regimes; however, there is no consensus on what characterises a fire regime. In this review article, the defining characteristics and drivers of fire regimes are explored and a new framework to understand fire regimes from a (bio)geographical perspective is proposed.

## Fire regime drivers and characteristics

Definitions of fire regimes vary widely with many authors finding it challenging to capture its essence. One prominent definition states that a fire regime is ‘… a structured description of the role of fire in ecosystems, mostly involving the parameterization of fire occurrence in a defined space–time window’ (Krebs et al. 2010:61). Oddi (2018:1) defines fire regimes as ‘the spatial and temporal pattern of fires and their effects in a given area and over a given time period’. Park and Allaby (2017) further expanded on this idea, suggesting that a fire regime can be understood as:

[*T*]he role that fire plays in an ecosystem, which depends on the frequency and scale of fires, and may include proposals for the controlled use of fire in a given area. (n.p.)

Table 1 presents the numerous characteristics that different authors have incorporated in their efforts to define the term ‘fire regime’. The majority of the authors specifically refer to frequency, seasonality, extent, intensity and type in their definitions. Additional characteristics include severity, burnt area, biomass, impacts and prescribed burning. Interestingly, ignition and fire weather are not prominently features in the definitions of the term. Davies (2013) contends that defining ‘fire regimes’ is a challenging and intricate task and points out that one must recognise that the components of fire regimes will vary in importance across different spatial and temporal scales, as well as among the biotic and abiotic elements of ecosystems.

**FIGURE 1:**
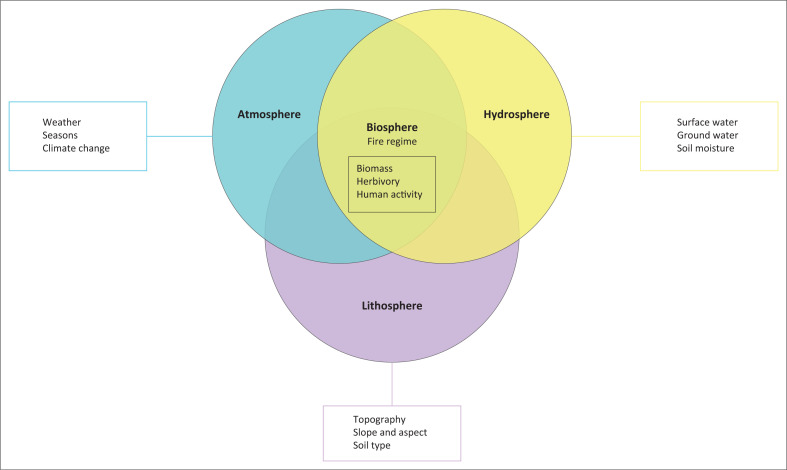
Biogeographical framework of fire regimes.

**TABLE 1:** Characteristics of fire regimes.

Author	Frequency	Seasonality	Extent or size	Intensity	Type	Severity	Burnt area	Biomass	Impacts	Prescribed burning	Ignition	Fire weather
Argañaraz et al. (2015)	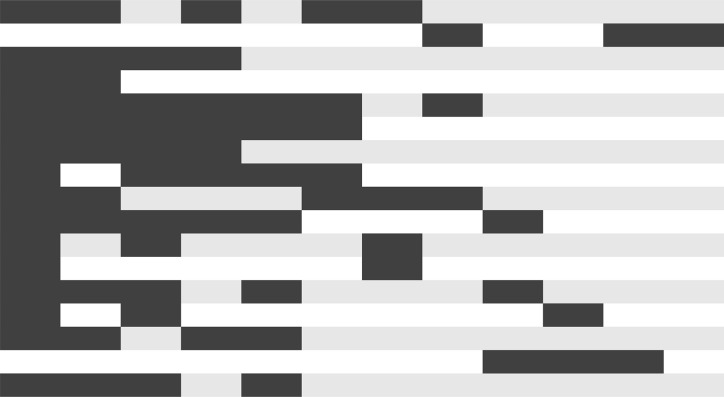											
Bedia et al. (2015)												
Braun de Torrez (2018)												
Chergui et al. (2018)												
Davies (2013)												
De Groot et al. (2013)												
Dye et al. (2020)												
Foster et al. (2020)												
Keeley & Syphard (2016)												
Krebs et al. (2010)												
Liu and Wemberly (2016)												
Meigs et al. (2018)												
Oddi (2018)												
Park and Allaby (2017)												
Pausas and Keeley (2014)												
Scheller et al. (2019)												
Whitman et al. (2015)												

The spatial distribution of fires is influenced by the interaction between climate-limited and fuel-limited fire landscapes, occasionally known as the aridity and productivity gradient (Prichard, Stevens-Rumann & Hessburg 2017). Climate-limited areas, which are highly productive in terms of vegetation and receive large quantities of rain – such as tropical rainforests – tend to have fewer fires. Conversely, arid regions like deserts, with minimal rainfall and low vegetation productivity, provide limited fuel for fires (Prichard et al. 2017). The influence of climate in fire regimes is a contentious issue, with some researchers arguing that the properties and amounts of biomass and human activities play a more prominent role in fire occurrences than climate (Archibald et al. 2018, Kraaij et al. 2018). Human activities affecting fire regimes mainly involve fire management practices and land use and land cover change (LULCC). Although it is essential to consider the biophysical variables of fire regimes, the influence of human actions has become increasingly apparent. Furthermore, Scheller et al. (2019) emphasise that contemporary fire regimes are predominantly shaped by human activities. Actions such as arson, deforestation, habitat fragmentation, introduction of invasive alien species, warfare, erection of artificial barriers, fuel treatments and patch burning can significantly modify fire regimes (Bowman et al. 2011). Since the 20th century, fire-suppression policies implemented by humans have led to the accumulation of fuel, thereby altering fire seasons (Batista et al. 2018; Holsinger et al. 2016).

## New framework

Numerous efforts have been made to generalise or simplify the factors that influence fires across different spatial and temporal scales. The literature is abundant with ‘fire triangle’ models developed by Moritz et al. (2005). While these attempts all start off with three requirements for a small fire to exist – oxygen, heat and fuel – they fail in the ‘succession’ of the requirements on a larger scale. For example, arguing that ignition only becomes important at ‘wildfire scale’ (Gomes, Miranda & Bustamante 2018) or ‘fire regime scale’ (Moritz et al. 2005) is odd as a source of ignition is required for microscale fires too. As the impact of humans on vegetation, climate and fire ignitions became more recognised, Bowman et al. (2011) introduced an evolved version of the fire triangle. This framework provides a more dynamic perspective on fire occurrences over time, incorporating elements such as climate change, agriculture, fire suppression and the domestication of plants, animals and fire. However, this representation entirely omits the role of topography. Archibald et al. (2008) developed a model to illustrate the factors leading to fire events in the southern African savannas. The unusual groupings of variables, particularly weather-related ones, presents challenges. For instance, wind speed affects not only fire intensity but also fire spread, and size and topography can influence wind speed and direction. Additionally, topography can influence wind speed and direction too. Recently Kelly et al. (2023) proposed a framework to understand fire regimes in general and in terms of the human impact thereof. Their basic model does not mention the hydrosphere at all and seem to indicate that fire regimes were virtually non-existent before human interference started during the Anthropocene. The hydrosphere’s role in fire regimes is often undervalued as water bodies not only serve as natural firebreaks but also as sources of soil moisture (Doerr & Shakesby 2013). Regions with significant soil moisture, such as stream catchments, are less prone to burning because the moisture must evaporate before the vegetation can ignite. Therefore, fires ignited in areas that experienced normal to above normal wet seasons are less likely to spread and the fire intensity will be low.

The proposed framework is built upon a classic representation of the Earth’s four spheres as shown in Figure 1. The rationale for this is to highlight the fact that fire events, and fire regimes, occur in the biosphere. The other three spheres – while simplistically depicted in the Figure 1, showing some of the components that play a role in fire – all influence conditions within the biosphere, where fire occurs. The role of spatial scale has often been underestimated in the previous attempts to conceptualise fire regimes. While one variable may be highlighted as the most prominent factor at microscale level, one still needs to grasp the interactions between various factors and how they ultimately influence the biomass (fuel) that is consumed by the fire.

The temporal aspects of fire regimes can be easily adapted to this framework as the varying micro to macroscale elements within each of the spheres are accommodated.

An argument may be made to extrapolate human activities from the figure as an independent entity from the biosphere because of the influence it has had on the Earth’s processes and functions. Kelly et al. (2023) argue that humans became the dominant driver in fire regimes at approximately 10 000 years before present. Yet, it is my argument that while one cannot deny the significant role humans have played in altering natural fire occurrences – human activities cannot be crowned as main drivers of contemporary fire regimes. Similarly, one should look at the broader scale and consider factors that are often over-looked in the climate versus fuel versus human activities debate, such as groundwater, soil moisture, soil type and herbivory. When considering the multitude of physical variables together with the interactions that humans have with them – which forms key to many (bio)geographical issues – one can achieve a more thorough understanding of a fire regime. Furthermore, there already exists a number of geographical processing tools and techniques such as remote sensing (RS) and geographic information systems (GIS), which are used to study fires (Alves & Perez-Cabello 2017; Argibay, Sparacino & Espindola 2020; Bar, Parida & Pandey 2020; Bui, Le & Hoang 2018; Colak and Sunar, 2020; Conedera et al. 2018; De Gouvenain, Midgley & Merow 2019; Kong et al., 2019; Ladbrook, Van Etten & Stock 2018; Long et al. 2019; Mayr, Vanselow & Samimi 2018; Nunes, Lourenço & Castro Meira 2016; Sannigrahi et al. 2020; Santana et al. 2018; Waigl et al. 2017). The main limitation of this approach is highlighting the potential of one discipline in understanding, planning and mitigating fire regimes while fire occurrence, spread, impacts and mitigation are transdisciplinary in nature.

## Conclusion

To predict the occurrence and spread of fire events, fires have been categorised into different fire regimes. A new framework for understanding fire regimes has been introduced, which incorporates often neglected variables such as soil moisture, soil type and herbivory. In addition, the framework aims to reduce the issues with accurately depicting scalar changes to variables from micro to macroscale. Currently, there is disagreement regarding the most significant driver of fires and fire regimes, with some authors pointing to the dominating role of humans over the environment. This contentious matter will need further exploration as fire regimes and regime shifts can differ within the same region. A biogeographical perspective of fire regimes offers an essential and comprehensive contribution to the fire science, the prediction of future fire events and disaster risk management. Further studies can explore the dimensions of the framework and its applications to specific fire-prone ecosystems.
